# Combined Effect of per- and Polyfluoroalkyl Substances, Toxic Metals, and Essential Elements on Chronic Kidney Disease

**DOI:** 10.3390/pollutants5020012

**Published:** 2025-05-13

**Authors:** Issah Haruna, Emmanuel Obeng-Gyasi

**Affiliations:** 1 Department of Built Environment, North Carolina A&T State University, Greensboro, NC 27411, USA; 2 Environmental Health and Disease Laboratory, North Carolina A&T State University, Greensboro, NC 27411, USA

**Keywords:** chronic kidney disease, per- and polyfluoroalkyl substances, toxic metals, essential elements, environmental mixtures, Bayesian Kernel Machine Regression, Weighted Quantile Sum

## Abstract

Chronic kidney disease (CKD) is a noteworthy global health issue affecting 10% of the world’s populace. It is increasingly linked to environmental exposures; however, the interplay of toxic metals, per- and polyfluoroalkyl substances (PFAS), and essential elements has not been fully elucidated. This cross-sectional study analyzed 5800 out of the 9245 participants from the 2017–2018 NHANES dataset to evaluate the combined effect of PFAS, essential elements, and toxic metals on CKD using logistic regression and advanced environmental mixture models, namely, Bayesian Kernel Machine Regression (BKMR), quantile g-computation (qgcomp), and Weighted Quantile Sum (WQS) regression. Our results showed cadmium (Cd) emerging as a significant contributor to CKD (OR = 2.16, *p* = 0.023) from the logistic regression analysis. Mercury (Hg) demonstrated the highest contribution in mixtures (posterior inclusion probability = 0.908) from our BKMR analysis, with a non-linear U-shaped dose–response relationship. Essential elements like selenium (Se) and manganese (Mn) exhibited protective correlations but complex non-linear interactions, moderating toxic metal effects from our qgcomp and WQS regression. Notably, antagonistic interactions between essential elements and some pollutants reduced the overall mixture impact on CKD, showing an overall decreasing joint effect of the combined PFAS, toxic metals, and essential elements on CKD, from the 25th to the 75th quantile. This study highlights the role of environmental co-exposures in CKD risk and highlights the need for advanced statistical and machine learning approaches in studying complex environmental mixture interactions on human health.

## 1. Introduction

Chronic kidney disease (CKD) is the progressive loss of kidney function over time, often resulting in end-stage renal disease (ESRD). Ten percent of the world population is affected, making it one of the most prevalent chronic conditions worldwide [1].

CKD imposes a huge burden on healthcare systems due to its association with cardiovascular disease, infections, and several comorbidities. While traditional risk factors, such as diabetes mellitus (DM), hypertension, and glomerulonephritis, are widely implicated, there is growing evidence that environmental factors, including exposure to chemicals, toxic metals, and imbalances in essential elements, play a pivotal role in CKD pathogenesis [2].

Among these environmental exposures, per- and polyfluoroalkyl substances (PFAS), a group of persistent organic pollutants, have received growing attention due to their widespread use, persistence in the environment, and bioaccumulation in human tissues [3]. Found in several products, including fire-extinguishing foams, non-stick pans, food packaging, and lubricants, PFAS enter the human body primarily through various sources, including contaminated food, water, and household dust [4]. Perfluorooctanoic acid (PFOA) and perfluorooctanesulfonic acid (PFOS) are two of the most studied PFAS, exhibiting significant nephrotoxicity [3]. Studies have shown that PFOA accumulates primarily in the kidneys, leading to impaired renal function [4]. Furthermore, sub-acute exposure to environmentally relevant concentrations of PFOA and GenX (a replacement compound for PFOA) in male guppies resulted in changes in testis transcriptome and reproductive traits, suggesting potential sublethal effects even at low concentrations [5]. The impact of PFAS on human kidney health is increasingly being examined as part of broader efforts to understand the multifaceted contributors to kidney disease [6].

In parallel, toxic metals like cadmium (Cd), lead (Pb), and mercury (Hg) represent another class of ubiquitous environmental pollutants that are nephrotoxic. These non-biodegradable metals tend to accumulate in the human tissues via ingestion, inhalation, and dermal absorption [7]. The kidneys, particularly the proximal tubules, serve as the primary targets for metal toxicity. Cd, for instance, accumulates in the proximal tubules of the kidney and is a well-established nephrotoxin. Chronic exposure to Cd has been associated with tubular dysfunction, proteinuria, and a decreased glomerular filtration rate (GFR) [8,9]. Similarly, Pb exposure is associated with nephrotoxic effects, including hypertension-related renal damage and chronic interstitial nephritis, even at low blood Pb levels [10]. These toxic metals exert their detrimental effects via inflammation, oxidative stress, and mitochondrial dysfunction, all of which contribute to kidney injury [11].

Prolonged exposure to these metals exacerbates renal deterioration and increases the risk of CKD development, particularly in regions with high environmental contamination [12].

While PFAS and toxic metals exert detrimental effects on renal function, essential elements such as iron (Fe), selenium (Se), and manganese (Mn) are vital for maintaining renal health. These trace elements support various physiological processes, including enzymatic functions, immune responses, and antioxidant defenses. Their relevance in renal health is underscored by their ability to mitigate oxidative stress and support cellular metabolism. For instance, Se is known to counteract the oxidative damage caused by Cd and other toxic metals, protecting renal tissues from injury [13]. However, imbalances—whether due to deficiencies or excess—can shift the scales towards toxicity. At elevated concentrations, Se, Mn, and Fe can promote ROS formation and renal tubular damage, exacerbating CKD progression [14,15]. Therefore, it is critical to explore the dualistic roles of these trace elements in a real-world exposure context using advanced statistical models to unravel their interplay on the influence on CKD risk within complex environmental mixtures.

Moreover, individuals with CKD may be at risk for both essential trace element deficiencies and toxicity due to the inability to excrete other non-essential elements, which can lead to accumulation within the kidney and cause chronic kidney damage, giving rise to hypertension, proteinuria, and disease manifestation. Environmental contamination stemming from industrial emissions, unsafe waste disposal, and agricultural runoff can further disrupt the mineral balance, increasing the burden of renal disease in exposed populations [16]. These exposures result in elevated concentrations of toxic metals and trace elements, contributing to nephrotoxicity or altered mineral homeostasis that impairs kidney function. Additionally, imbalances in essential elements, whether due to deficiency or excess, can adversely affect kidney function [17]. For instance, Se deficiency has been linked to increased susceptibility to renal oxidative damage, while excessive levels can induce cytotoxic effects in renal cells through the production of reactive oxygen species [15,18]. Therefore, exploring the relationship among PFAS, essential elements, and toxic metals is critical for identifying protective strategies against metal-induced nephrotoxicity.

The interplay among PFAS, toxic metals, and essential elements may have synergistic or antagonistic effects on CKD risk. For example, sufficient Se and Mn levels have been shown to mitigate Cd-induced nephrotoxicity, while concurrent exposure to Pb and Cd can amplify renal injury [2,11]. These complex interactions are further influenced by factors such as genetic predisposition, nutritional status, and co-existing comorbidities, making it critical to study these exposures in a holistic manner.

Despite significant advances, there remains a critical gap in comprehensively elucidating the joint impact of PFAS, essential elements, and toxic metals on CKD development. This gap is particularly urgent in low- and middle-income countries, where exposures are often higher due to inadequate regulation, and nutritional deficiencies are more prevalent [1]. The primary objective of this study, therefore, is to assess the combined and potentially interactive effects of PFAS, toxic metals, and essential elements on kidney health using advanced statistical approaches designed to characterize the complex, often non-linear relationships inherent in real-world exposure mixtures. Addressing these knowledge gaps is essential for identifying at-risk populations and developing targeted preventive strategies and public health policies to reduce the incidence and prevalence of the disease.

## 2. Materials and Methods

### 2.1. Study Population

This cross-sectional analysis utilized data from a subset of the 2017–2018 cycle of the National Health and Nutrition Examination Survey (NHANES), a multistage, stratified study designed to provide comprehensive insights into the general health of a nationally representative population of non-institutionalized individuals in the United States. This survey is unique in that it integrates both interviews and clinical assessments to gather data on the prevalence of chronic and infectious diseases, including those that may be undiagnosed. The NHANES also collects data on risk factors such as obesity, hypertension, alcohol intake, serum cholesterol levels, toxic chemical levels in blood and urine, and nutritional and dietary status. All participants provided informed consent prior to participation. The study protocols were approved by the Institutional Review Board of the National Center for Health Statistics (NCHS), which operates under the Centers for Disease Control and Prevention (CDC), under protocols #2011–2017 and #2018–01. Our team used deidentified data, which were determined to be from nonhuman subject research by the Office of Research Compliance and Ethics at North Carolina Agricultural and Technical State University. Out of the 12,254 participants, only 5800 were used in our analysis due to the availability of relevant data in determining the presence or absence of CKD.

### 2.2. PFAS Extraction and Quantitation

This study focused on two specific PFAS: PFOA and PFOS. Blood sample analysis for these compounds involved a detailed quantification process. The measured analytes included the total of monomethyl-branched PFOS isomers (Sm-PFOS), linear PFOA (n-PFOA), linear PFOS (n-PFOS), and the total of branched PFOA isomers (Sb-PFOA). Detection and measurement were conducted using an advanced method combining solid-phase extraction integrated with high-performance liquid chromatography (HPLC) analysis, followed by turboionspray ionization and tandem mass spectrometry (SPE-HPLC-TIS-MS/MS). Shortly after dilution with formic acid, 50 μL of the serum sample was introduced into a commercially available column-switching system. This ensures the analytes are concentrated on the solid-phase extraction column. Separation of the analytes from each other and from other serum components was carried out using HPLC. PFAS detection and quantification were carried out using tandem mass spectrometry paired with negative-ion turboionspray ionization, allowing for high sensitivity with detection limits in the low nanograms per milliliter (ng/mL) range. This method enables efficient and accurate measurement of PFAS concentrations in serum, following established analytical protocols [19]. When the measured concentrations of analytes fell below the lower limit of detection (LLOD), a substituted value was used in the dataset. This imputed value was calculated by dividing the LLOD by the square root of two (LLOD/√2). For both PFOA and PFOS, the LLOD was established at 0.10 ng/mL.

### 2.3. Quantification of Lead, Cadmium, and Mercury Alongside Selenium, Manganese, and Iron

To assess concentrations of heavy metals and essential elements in blood samples from the NHANES 2017–2018 dataset, the National Center for Environmental Health at the CDC utilized ICP-DRC-MS (inductively coupled plasma–dynamic reaction cell–mass spectrometry) for analysis. This technique was used to quantify levels of Pb, Cd, and Hg, as well as the essential elements Mn and Se. This method directly measures the concentration of each metal in whole-blood samples collected in EDTA tubes (from BD Bioscience, Franklin Lakes, NJ, USA) to prevent coagulation and ensure uniform distribution of blood components using Agilent 7900 inductively coupled plasma–dynamic reaction cell–mass spectrometry (ICP-DRC-MS) after 1:50 dilution with the reagent mix that solubilizes blood components and releases metals. A high-temperature argon plasma (6000–8000 K) ionizes the metal atoms, and the metal ions pass through the ion pass through a dynamic reaction cell to reduce interference and then through the quadrupole mass filter and are detected at the electron multiplier detector for quantification. Rhodium, tellurium, and iridium were used as internal standards to correct for matrix suppression and instrument drift to ensure accuracy in measurement. The data are processed and analyzed using Agilent Mass Hunt software (version B.07.00), which calculates the concentration of metals based on calibration curves to ensure reproducibility and accuracy. The NHANES website includes extensive laboratory protocols for quality control and assurance data [20]. Fe was measured using the Roche Cobas 6000 analyzer (c501 module) with a colorimetric method involving FerroZine reagent. In this method, Fe^3+^ is released from transferrin, reduced to Fe^2+^ by ascorbate, and reacts with FerroZine to form a colored complex. The absorbance of this complex, measured at 546 nm, is directly proportional to the iron concentration. The samples with concentrations falling below the limits of detection (LODs) were substituted with a value calculated as the LOD divided by the square root of 2 (LOD/√2) to minimize bias. The detection limits for the metals assessed in this analysis were as follows: Pb (0.07 μg/dL), Cd (0.10 μg/L), total Hg (0.28 μg/L), Se (24.48 μg/L), Mn (0.99 μg/L), and Fe (5 μg/dL).

### 2.4. CKD Biomarkers

#### 2.4.1. Ratio of Urinary Albumin to Creatinine

In the 2017–2018 NHANES, the urine albumin-to-creatinine ratio (UACR) was calculated as such: UACR = urine albumin/urine creatinine ×100, round to 0.01 [21], albumin in urine was measured in micrograms per milliliter (μg/mL), and creatinine in urine in milligrams per liter (mg/L). Albuminuria was classified into two categories based on albumin-to-creatine ration values: microalbuminuria (30–300 mg/g) and macroalbuminuria (>300 mg/g).

#### 2.4.2. Estimated Glomerular Filtration Rate (eGFR) and CKD

The estimated glomerular filtration rate (eGFR) was determined using the equation developed by the Modification of Diet in Renal Disease (MDRD) study [22,23]. The formula used was:

$$\text{eGFR}\left(\text{mL}/\text{min}/1.73\;{\text{m}}^{2}\right)=175\times {\left(\text{Scr}\right)}^{-1.154}\times {\left(\text{Age}\right)}^{-0.203}\times (0.742\;\text{if}\;\text{female})\times (1.210\;\text{if}\;\text{African}\;\text{American}).$$

where Scr represents serum creatinine (mg/dL).

Kidney function was then classified into five categories based on eGFR values, following the staging criteria provided by the National Kidney Foundation and supported by Babekir et al. [24,25], with eGFR measured in mL/min/1.73 m^2^:

Stage 1: eGFR ≥ 90.
Stage 2: eGFR from 60 and 89.
Stage 3: eGFR ranging from 30 and 59.
Stage 4: eGFR ranging from 15 and 29.
Stage 5: eGFR < 15.

CKD status was classified as either present or absent, with the “present” category including all CKD stages based on the criteria established by the American Kidney Fund [26].

### 2.5. Selected Covariates and Variables for Analytical Adjustment

The outcome variable in this study was CKD. The main predictor variables included PFAS (PFOA and PFOS), essential elements (Se, Mn, and Fe), and metal exposure (Cd, Pb, and Hg). The variables included for model adjustment were age, sex, race/ethnicity, smoking status, body mass index (BMI), alcohol consumption, diabetes status, annual income, and hypertension. These covariates were selected based on prior literature examining the impact of environmental pollutants, particularly metals, on renal health [27,28].

### 2.6. Statistical Analysis

#### 2.6.1. Summary Statistics, Regression, Correlations, and Statistical Testing

This study initially used descriptive statistics to assess the demographic characteristics and exposures in the dataset. Spearman correlation analysis was performed to evaluate the interrelationships among pollutants. Additionally, logistic regression was employed to examine the associations between pollutant exposures and CKD, as well as to assess their statistical significance. Due to the data not being normally distributed, we employed the Mann–Whitney U test, a non-parametric statistical alternative for the *t*-test when the data are not normally distributed. The chi-square test was also conducted to determine if there was statistical significance between the categorical exposure variable and CKD outcome. To maintain analytical integrity, missing data for key variables were imputed using median values. This approach allowed for a more complete dataset and helped reduced potential bias associated with missing information.

#### 2.6.2. Bayesian Kernel Machine Regression

In this study, we used Bayesian Kernel Machine Regression (BKMR), as described by Bobb et al. [29], and implemented the Markov Chain Monte Carlo (MCMC) sampling approach with 5000 iterations.

For each subject *i* = 1,…, *n*, the model is defined as:

$$\text{Yi}=\text{h}(\text{zi})+{\text{Xi}}^{\text{T}}\beta +\epsilon_{\text{i}}$$

where *Yi* is a health endpoint, z_i_ = (z_i1_,…, z_iM_)^T^ is a vector of *M* exposure variables (such as metals’ constituents), **x**_i_ represents a vector of covariates (potential confounders), ϵ_i_ ~N(0,σ^2^) is an error term, and h(·) is an unknown, potentially non-linear and non-additive function estimated using kernel machine methods. BKMR is well suited for evaluating complex environmental mixtures, as it flexibly captures non-linear effects and interactions among mixture components without requiring explicit specification of the functional form of h(·). The BKMR analysis brought forth posterior inclusion probabilities (PIPs), which are crucial in assessing the importance of individual PFAS, individual essential elements, and toxic metals in the mixture. The PIPs, which ranged between zero and one, aided in determining the relative importance of each constituent in the mixture [30]. To further explore the exposure–response relationship, we evaluated the estimated function h(z) across varying levels of specific exposures, while fixing the other exposures at their median value [31]. This approach allowed us to visualize how individual components contribute to CKD risk within the broader mixture context.

The BKMR model was particularly valuable in our analysis due to its ability to generate intuitive graphical outputs. This feature facilitated the comparison of individual and joint exposure effects by contrasting outcomes at specific exposure percentiles with those at the median levels. Through this approach, we gained deeper insight into the distinct and combined influences of PFOA, PFOS, toxic metals (Pb, Cd, Hg), and essential elements (Se, Mn, Fe) on CKD.

#### 2.6.3. Quantile g-Computation

We employed quantile g-computation (qgcomp), a novel statistical method designed to estimate the joint effects of exposure mixtures. This method mixes the inferential straightforwardness of Weighted Quantile Sum (WQS) regression with the adaptability of g-computation, a technique for estimating causal effects [32]. G-computation is a statistical modeling technique used to estimate the joint effect of simultaneous, quantile-level increases in all components of an exposure mixture. Specifically, it provides an estimate of the expected change in the outcome associated with a one-quantile increase in all exposures, holding covariates constant. Unlike Weighted Quantile Sum (WQS) regression, which assumes directional homogeneity and uses pre-specified constraints, qgcomp allows for exposures to have effects in different directions and yields unbiased effect estimates with valid confidence interval coverage, even under moderate sample sizes typical of epidemiological studies [33].

The general equation is given by

$$\text{Yi}=\beta 0+\sum_{J=1}^{d}\beta \text{jXqji}+\epsilon \text{i}$$

where Yi = outcome variable individual I, β0 = intercept, d = number of exposure variables in the mixture, Xqji = quantile-transformed exposure variable j for individual i, βj = estimated regression coefficient for each quantized exposure j, capturing its individual contribution, and ϵi = error term.

#### 2.6.4. Weighted Quantile Sum Regression

WQS regression is a commonly used method in environmental epidemiology to assess the joint effect of chemical mixtures on health outcomes. In this study, we applied WQS regression to estimate the combined effect of PFOA, PFOS, Cd, Pb, Hg, Se, Mn, and Fe on the risk of chronic kidney disease (CKD). To reduce the influence of sample-specific weights, the data were partitioned into a training and test set, as recommended in prior applications [34]. The general structure of the WQS generalized non-linear regression model, applied to a mixture containing c components, is expressed as follows:

$$g\left(u\right)=\beta 0+\beta 1\left(\sum_{i=1}^{c}\text{wiqi}\right)+\text{z}\prime \Phi$$

In this equation, g(u) denotes the link function connecting the mean (μ) to the predictors, consistent with generalized linear model frameworks. The term *β*0 denotes the intercept, while *β*1 corresponds to the coefficient of the WQS index. The weights *wi*, which reflect the contribution of each mixture component, are estimated through an ensemble approach using either bootstrap resampling or random subset sampling. The *qi* terms are the quantile-scored values of each component, and *z*’*ϕ* represents the vector of covariates and their associated parameters.

The estimated weights are subject to the following constraints: the sum of all weights (∑*w*_i_) must equal 1, and each individual weight must fall within the range of 0 to 1 (0 ≤ *w*_i_ ≤ 1). Once weights are determined for each ensemble sample, the WQS index is calculated using the formula $\text{WQS}=\left(\sum_{i=1}^{c}\text{wiqi}\right)$, where *w*_i_ represents the average weight across the ensemble iterations. These weights are linked to either a positive or negative *β*_1_, depending on the direction specified for the relationship between the mixture and the outcome.

Our study’s analysis was conducted with R (version 4.2.3; R Foundation for Statistical Computing, Vienna, Austria) [35]. The significance level was set at 0.05 for non-Bayesian analysis, with all analyses adjusted for the covariates, BMI, gender, age, annual income, alcohol intake, smoking, and ethnicity.

## 3. Results

### 3.1. Demographic, Exposure, and Clinical Characteristics

This study leveraged data from 5800 NHANES study participants to explore the combined effects of PFAS, toxic metals, and essential elements of CKD within a diverse population (Table 1). Out of the total number of participants, 1071 (18.47%) presented with CKD, while 4729 (81.53%) did not have CKD. This study also revealed significant socioeconomic, lifestyle, and clinical differences between the two groups, providing information on associated risk factors and contributors of CKD.

Additionally, the Mann–Whitney U test revealed a significant association between CKD and PFOS (*p* = 0.0004), Pb (*p* < 0.0001), Cd (*p* < 0.0001), Fe (*p* < 0.0001), eGFR (*p* < 0.0001), ACR (*p* < 0.0001), and Scr (*p* < 0.0001).

### 3.2. Assessing Relationships Among Key Variables: Spearman Pearson Correlation Matrix Analysis

**Figure 1.**
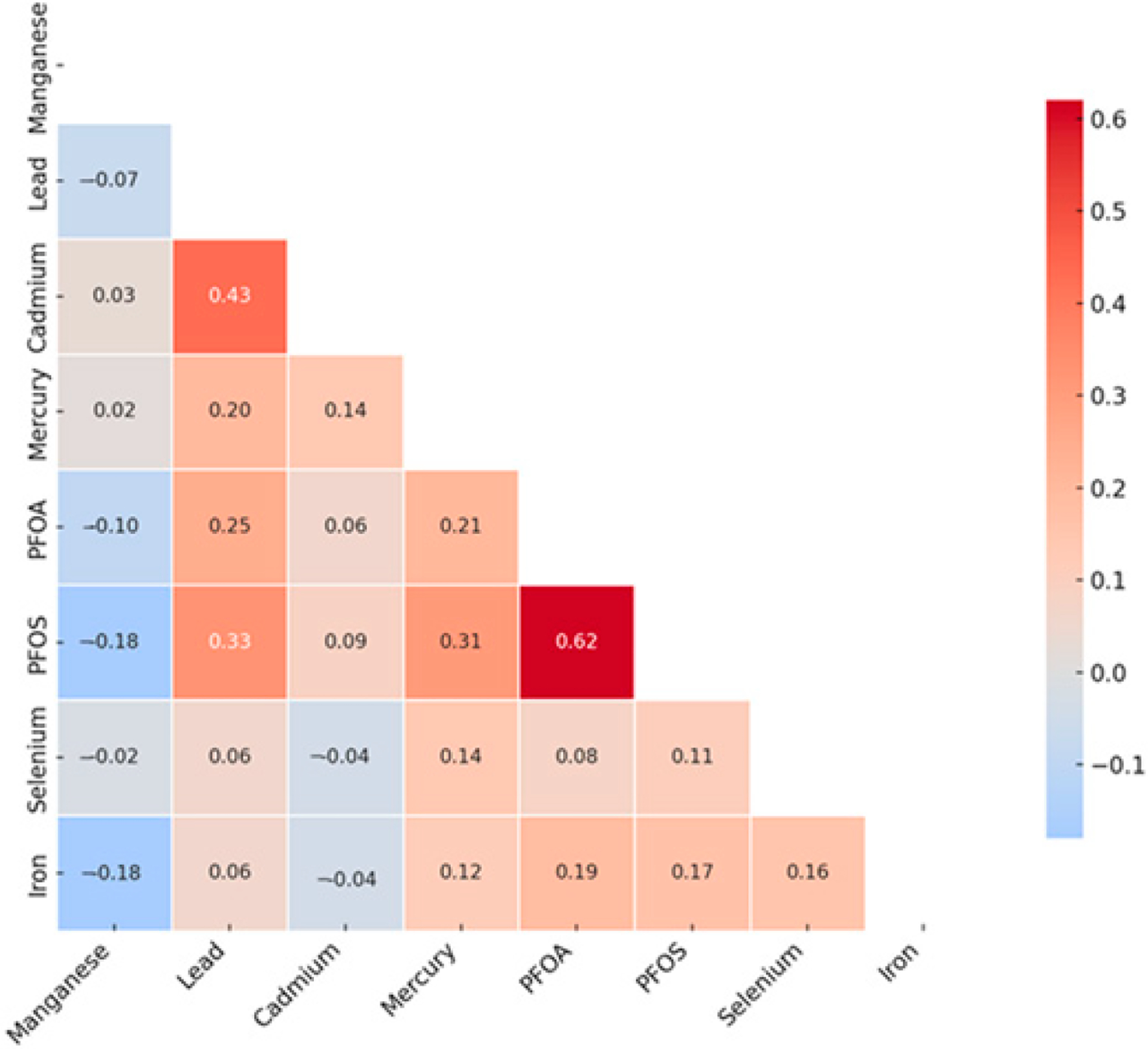
Spearman correlation analysis of toxic metals and essential elements with CKD. PFOA: perfluorooctanoic acid, PFOS: perfluorooctanesulfonic acid.

The Spearman correlation heatmap (Figure 1) revealed several notable pairwise relationships among the toxic metals, essential elements, and PFAS compounds included in this study. The strongest positive correlation was observed between PFOA and PFOS (ρ = 0.62), suggesting a potential shared environmental source or similar exposure pathways. A moderate positive correlation was also observed between Pb and Cd (ρ = 0.43), which may reflect overlapping exposure sources, such as industrial emissions, contaminated soil, or tobacco smoke. Additional moderate correlations included PFOS with Pb (ρ = 0.33) and PFOS with mercury (Hg) (ρ = 0.31). In contrast, weak negative correlations were noted between manganese (Mn) and PFOS (ρ = −0.18), Mn and PFOA (ρ = −0.10), and selenium (Se) and Cd (ρ = −0.04), potentially reflecting divergent environmental sources, biological functions, or metabolic behavior.

### 3.3. Logistic Regression Analysis of Exposure Variables with CKD

To evaluate the association between environmental exposures and CKD risk, logistic regression analysis was conducted on key exposure variables. The results show that PFOA (OR = 1.37, *p* = 0.039) and Cd (OR = 2.161, *p* = 0.0023) were significantly associated with an increased risk of CKD. The full results are presented in Table 2.

### 3.4. BKMR

#### 3.4.1. Evaluating PFAS and Metals’ Contributions to CKD Using BKMR

Table 3 presents the PIPs for the exposure variables with CKD. The PIP quantifies the relative importance of each exposure in contributing to CKD, with higher values indicating more importance.

Table 4 shows the hierarchical PIP analysis, which explores the interplay among the PFAS, toxic metals, and essential elements of CKD utilizing the group-wise Bayesian framework. The exposure variables are grouped into three categories based on having similar characteristics. Group 1, consisting of PFOA and PFOS, exhibited the lowest group-level PIP (groupPIP = 0.251), indicating that PFAS collectively have weaker relevance to CKD. PFOA showed the highest conditional PIP (Cond PIP = 0.578) in this group, affirming its importance within PFAS. For group 2, consisting of Pb, Cd, and Hg, the overall groupPIP was =0.684, indicating that among the exposure groups, it had the second highest relevance to CKD. Conditional PIP results found that Hg (condPIP = 0.855) emerged as the most notable contributor. Group 3, made up of Se, Mn, and Fe, had the largest groupPIP of 0.702. Among them, Fe (condPIP = 0.835) emerged as the highest contributor.

#### 3.4.2. Univariate Analysis of Individual Effects of PFAS, Toxic Metals, and Essential Elements on CKD

**Figure 2.**
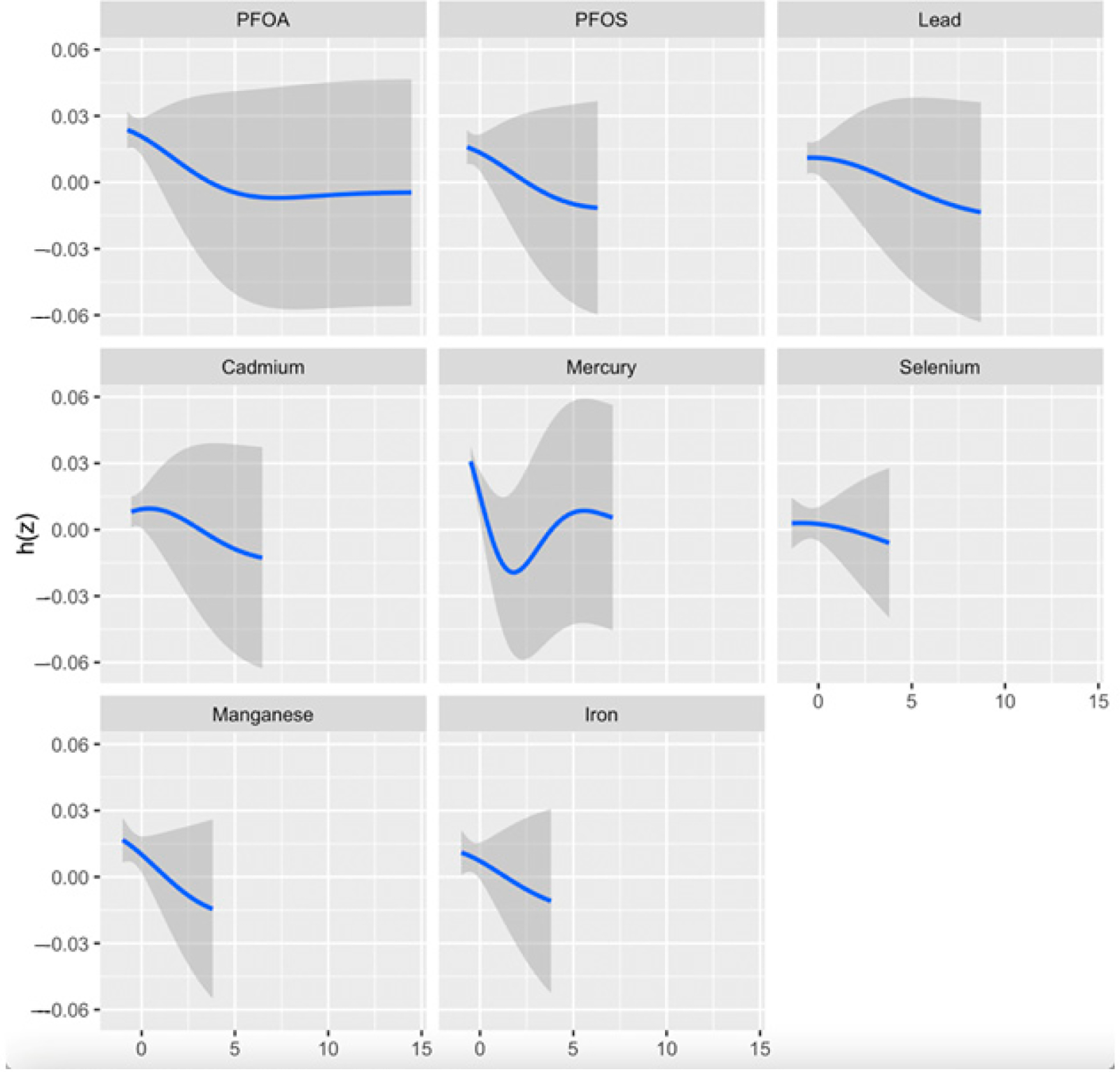
Univariate exposure–response and 95-percent credible interval (gray area) for each toxic metal and essential element when all other exposures are fixed at the 50th quantile. Adjusted for age, gender, BMI, ethnicity, alcohol intake, smoking, hypertension, diabetes, and income. PFOA: perfluorooctanoic acid, PFOS: perfluorooctanesulfonic acid; h(z); estimated exposure–response.

The univariate exposure–response analysis in BKMR provides a visual assessment of how each exposure—PFOA, PFOS, Hg, Cd, Pb, Fe, Se, and Mn—is individually associated with CKD, while holding all other exposures in the mixture at their median values. As shown in Figure 2, this approach allows for the identification of potential non-linear relationships. Notably, the plot suggests a non-linear association between Hg and CKD risk.

#### 3.4.3. Bivariate Exposure–Response Functions with Fixed Quantile Values

**Figure 3.**
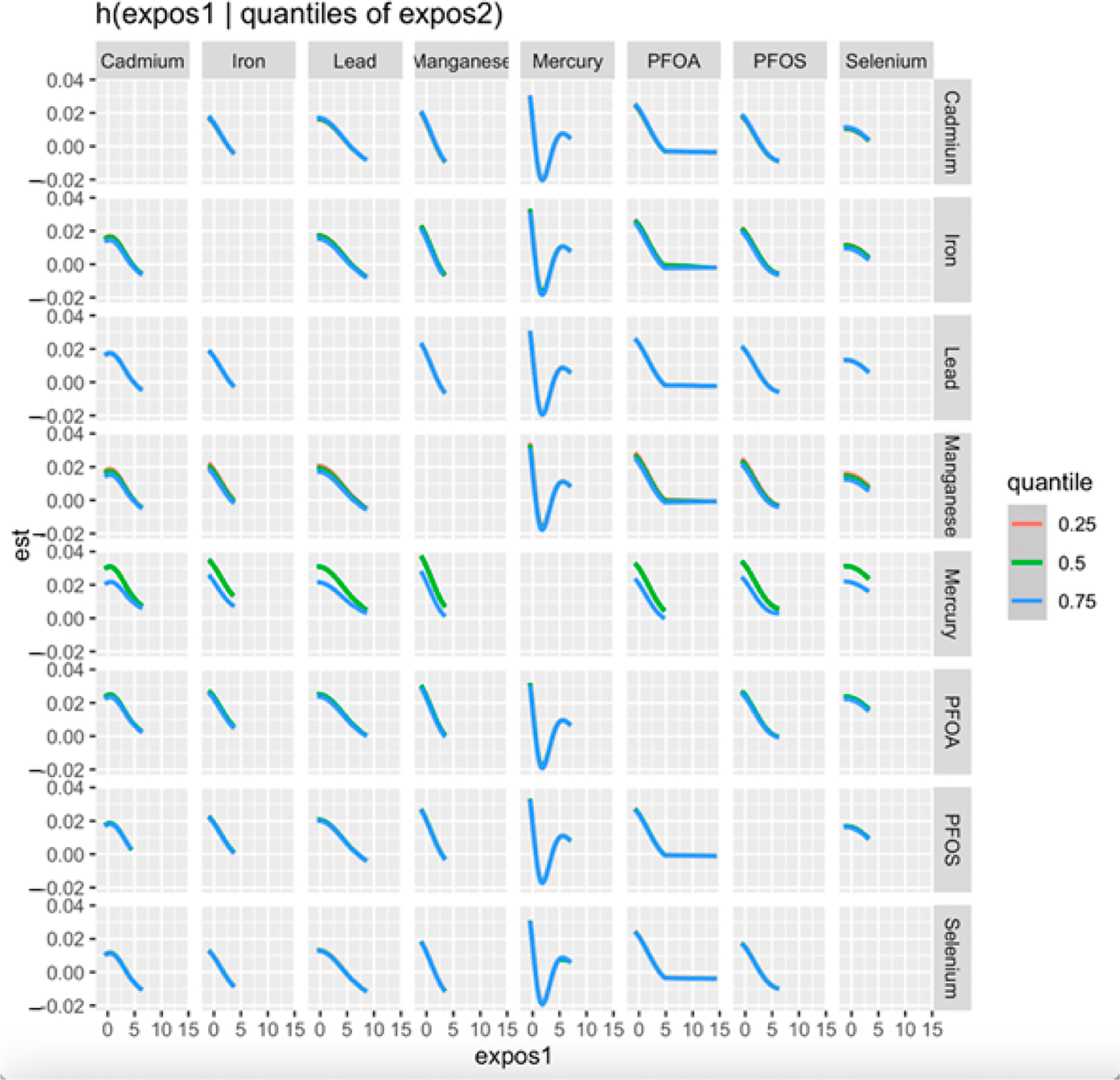
Bivariate exposure–response for each toxic metal and essential element with the first exposure (x-axis) increasing from left to right and second exposure fixed at the 0.25, 0.50, and 0.75 quantiles while all other exposures are fixed at the 50th quantile. Adjusted for age, gender, ethnicity, alcohol intake, smoking, hypertension, diabetes, and income. PFOA: perfluorooctanoic acid, PFOS: perfluorooctanesulfonic acid.

Figure 3 displays the association between a progressively increasing level of one pollutant and CKD while the second pollutant was fixed at the 0.25 (red line), 0.5 (green line), and 0.75 (blue line) quantiles. The models were adjusted for relevant covariates, including age, gender, BMI, income, ethnicity, diabetes, smoking status, alcohol use, and hypertension. The x-axis, labeled as “expos1”, depicts the levels of one exposure, whereas the y-axis, designated as “est”, shows the estimated effect on CKD.

#### 3.4.4. Single-Variable Effects of PFAS, Toxic Metals, and Essential Elements on CKD

**Figure 4.**
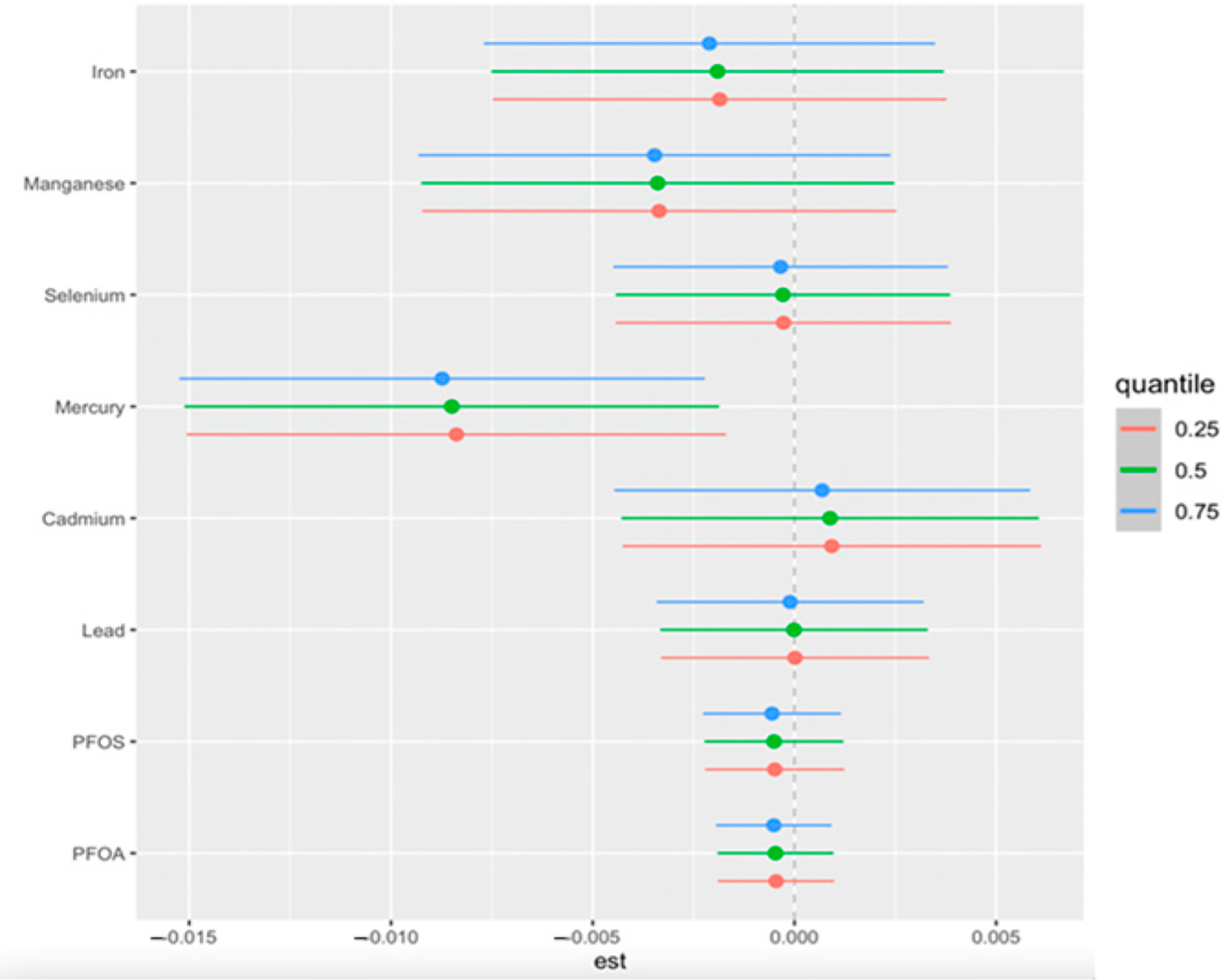
Single-exposure effect of the individual exposure on CKD (estimates and 95% credible interval) examining the change in response associated with a change in a single exposure from its 25th to 75th quantile while all other exposures are fixed at a specific quantile (25th, 50th, and 75th). Adjusted for age, gender, ethnicity, alcohol intake, smoking, hypertension, diabetes, and annual income. PFOA: perfluorooctanoic acid, PFOS: perfluorooctanesulfonic acid.

We conducted single-exposure analyses while adjusting for key demographic and clinical covariates to assess the independent effects of each environmental exposure on CKD risk. This approach allows us to examine how changes in individual exposures, from their 25th to 75th percentile, influence CKD risk while holding all other exposures constant at specific percentiles (25th, 50th, and 75th). The estimates, along with their 95% credible intervals, are displayed in Figure 4.

#### 3.4.5. Single-Exposure Interaction Terms of PFAS, Essential Elements, and Toxic Metals

**Figure 5.**
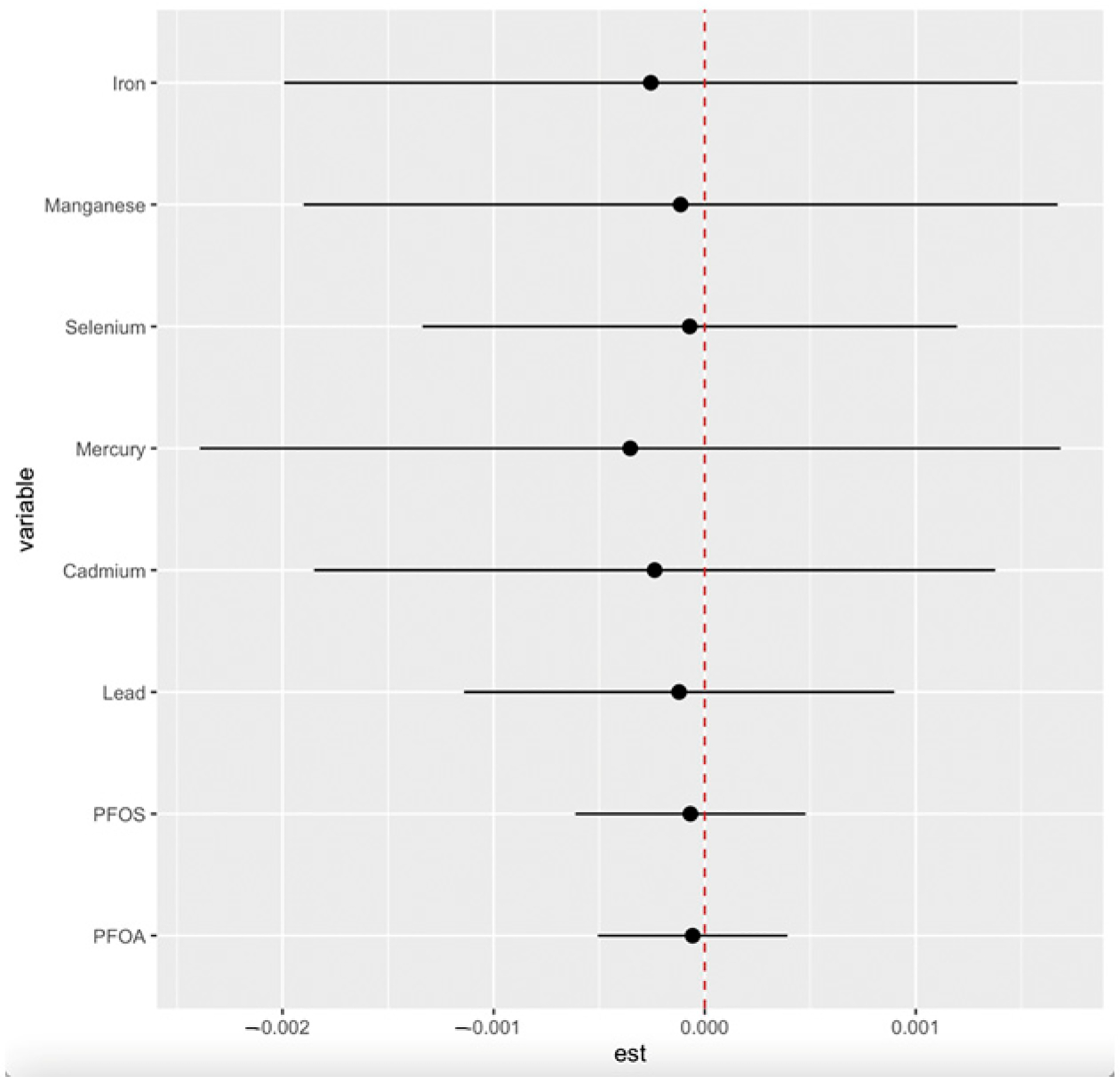
Single-exposure interaction terms of PFAS, toxic metals, and essential elements, comparing the effect of each exposure (from its 25th to 75th percentile) when all others are fixed at the 25th percentile compared to the 75th percentile. Adjusted for age, gender, ethnicity, alcohol intake, smoking, hypertension, diabetes, and annual income. PFOA: perfluorooctanoic acid, PFOS: perfluorooctanesulfonic acid.

Beyond single-exposure analyses, we examined potential interactions between PFAS, toxic metals, and essential elements by comparing the effects of each exposure when all others were fixed at the 75th quantile versus the 25th quantile. This approach provides insight into how environmental exposure to a single pollutant interacts with multiple pollutants together. Figure 5 presents the estimated interaction terms for each exposure, adjusted for key demographic and clinical confounders, including age, gender, ethnicity, alcohol intake, smoking, hypertension, diabetes, and annual income.

#### 3.4.6. Overall Effects of PFAS, Toxic Metals, and Essential Elements on CKD

**Figure 6.**
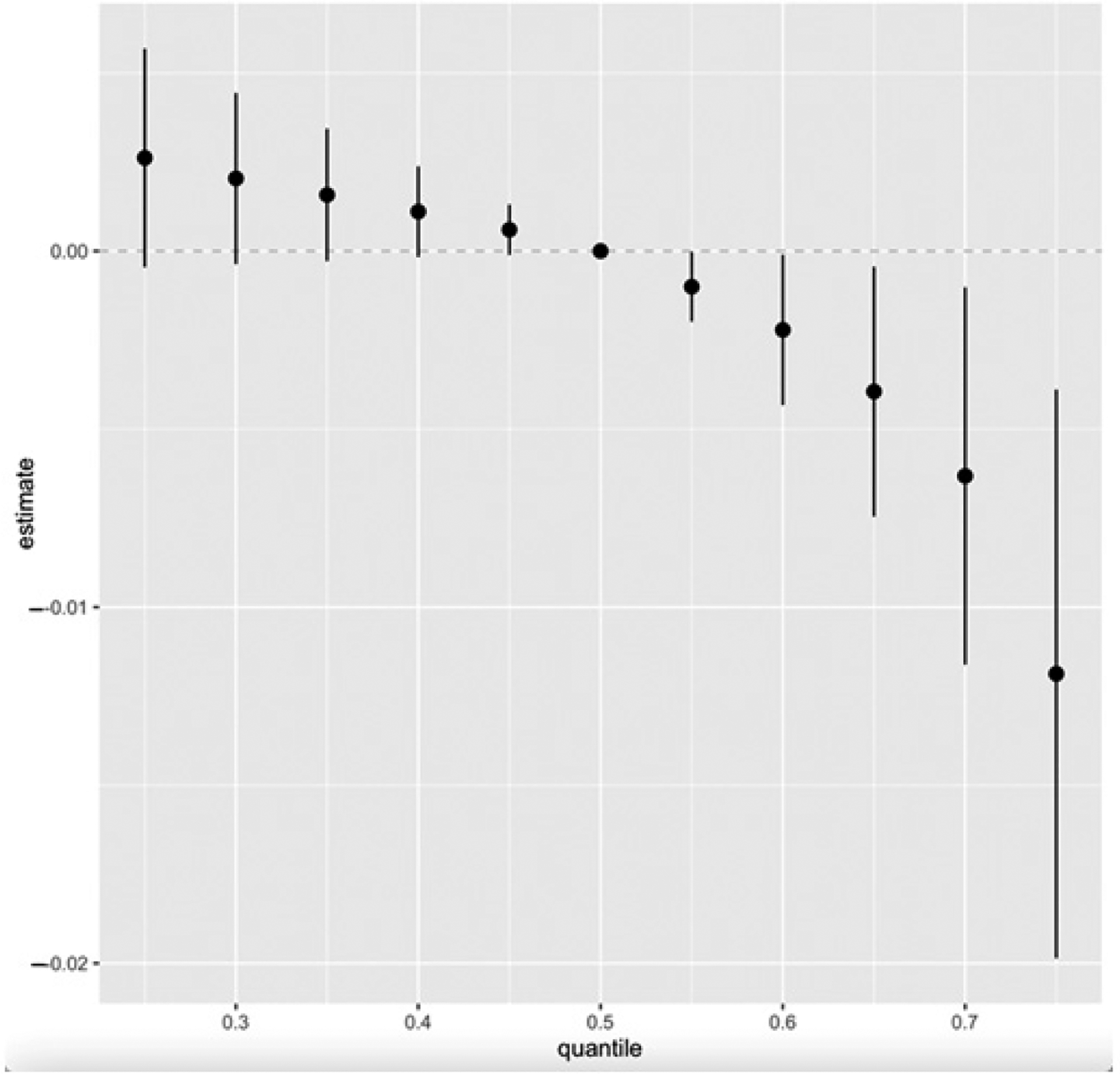
Overall exposure effect showing all exposures increasing from the 25th to the 75th percentile as compared to the 50th percentile. The overall effect of exposure mixtures on CKD (estimates and 95% CI). Adjusted for age, gender, alcohol intake, smoking, hypertension, diabetes, and annual income.

Figure 6 presents the cumulative effect of the exposure mixture on CKD. Exposure levels were set at various quantiles between the 25th and 75th, increasing in five-point increments. The median value (50th quantile) is used as the reference point for comparison across these exposure levels.

### 3.5. Quantile g-Computation

**Figure 7.**
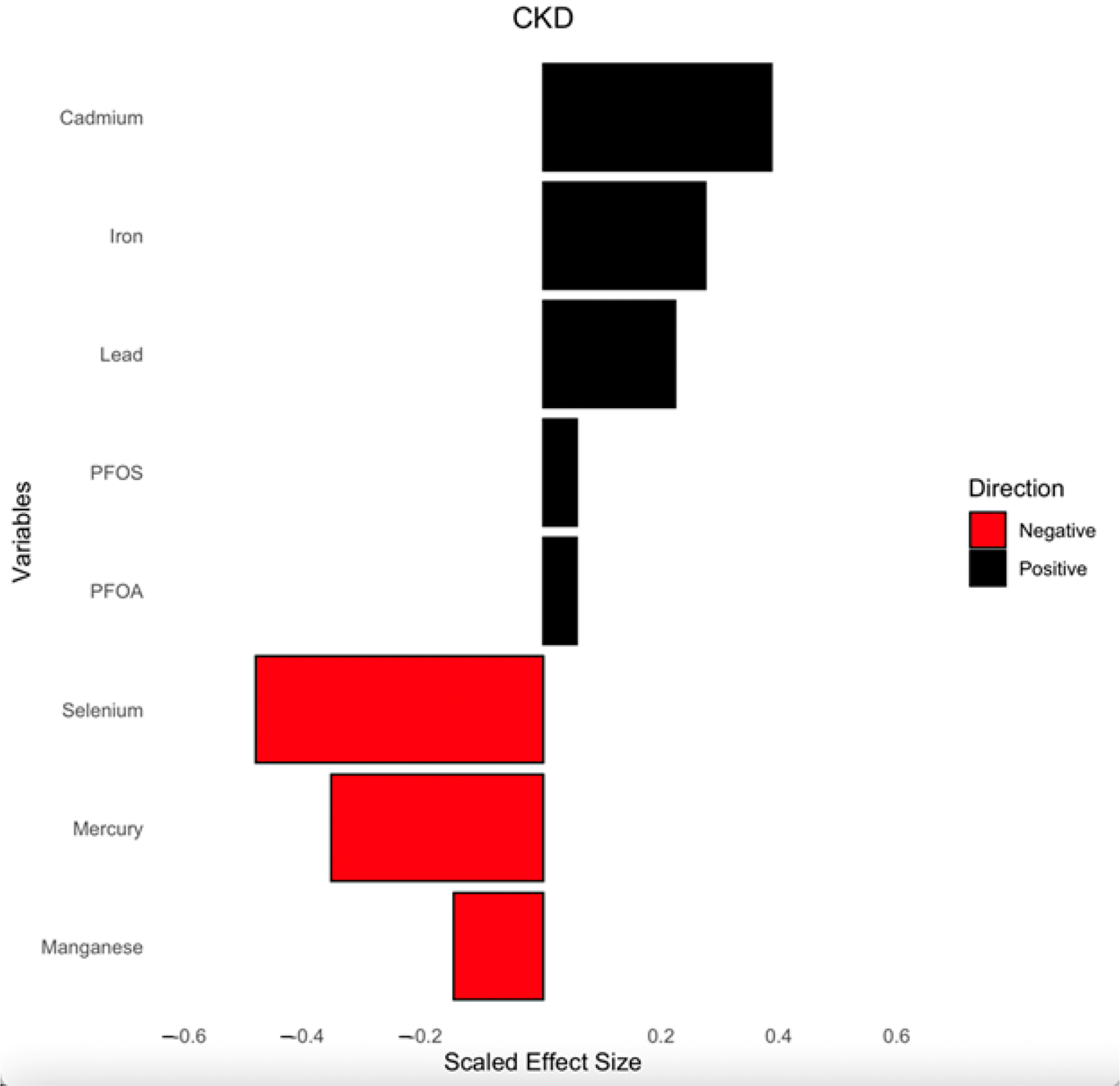
Weights from qgcomp regression model for the effects of the mixture of PFAS, toxic metals, and essential elements on CKD. Analysis was adjusted for age, gender, ethnicity, alcohol intake, smoking, hypertension, diabetes, and annual income. PFOA: perfluorooctanoic acid, PFOS: perfluorooctanesulfonic acid.

Figure 7 presents the weights derived from the qgcomp model, illustrating the relative contributions of PFAS, toxic metals, and essential elements to CKD risk. The analysis accounts for key demographic and clinical confounders, including age, gender, ethnicity, alcohol intake, smoking, hypertension, diabetes, and annual income.

The direction and magnitude of each exposure’s effect are represented by bars, with black bars indicating positive associations with CKD and red bars representing negative associations. Cd, Fe, Pb, PFOS, and PFOA showed positive associations, suggesting they may contribute to increased CKD risk. Conversely, Se, Hg, and Mn displayed negative weights, implying a potential protective or inverse relationship with CKD risk. The scaled effect sizes help contextualize the relative importance of each exposure within the mixture.

This analysis provides insight into how multiple environmental exposures collectively influence CKD risk and the impact of individual pollutants within that mixture.

### 3.6. Weighted Quantile Sum (WQS) Regression

**Figure 8.**
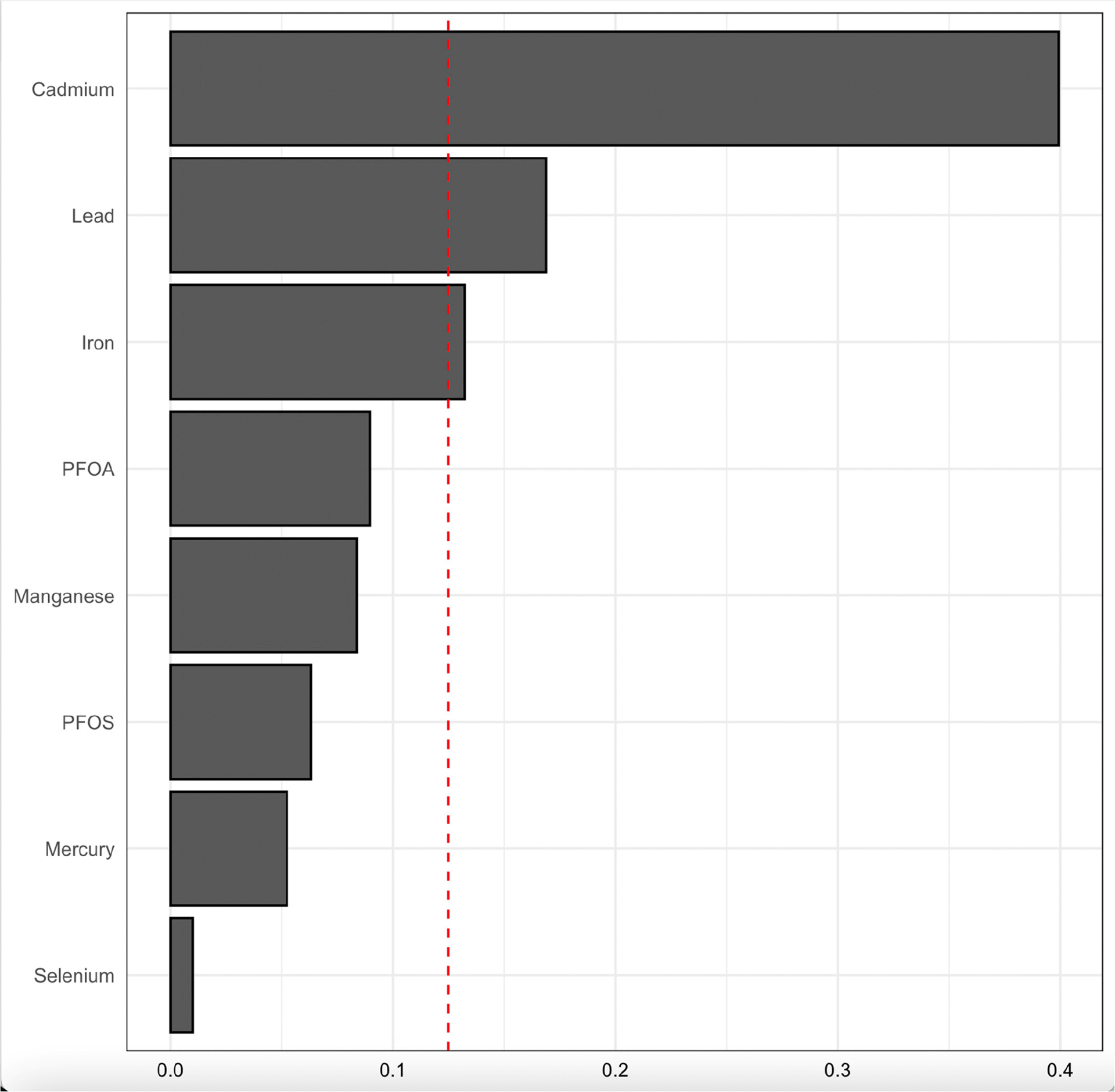
WQS regression model for contributions of blood concentrations of PFAS, toxic metals, and essential elements to CKD outcome. The model was adjusted for age, gender, ethnicity, alcohol intake, smoking, hypertension, diabetes, and annual income. PFOA: perfluorooctanoic acid, PFOS: perfluorooctanesulfonic acid.

Figure 8 displays the WQS regression model results, illustrating the relative contribution of blood concentrations of PFAS, toxic metals, and essential elements to CKD risk. The model accounts for key demographic and clinical confounders, including age, gender, ethnicity, alcohol intake, smoking, hypertension, diabetes, and annual income.

The bars represent the weights assigned to each exposure, indicating their relative importance in the mixture’s overall effect on CKD risk. Cd, Pb, and Fe had the highest weights, suggesting a strong contribution to CKD risk, while PFOA, Mn, PFOS, Hg, and Se exhibited lower relative contributions; however, their effect was notable (non-zero). The red dashed line provides a reference threshold, highlighting exposures with weights that may be particularly influential in the model.

## 4. Discussion

### 4.1. Environmental Pollutants, Essential Elements, and Kidney Disease: Unraveling Complex Interactions

The global burden of CKD underscores the urgent need to elucidate the multifactorial contributors to renal dysfunction, especially in environmentally vulnerable populations. This study leveraged a nationally representative survey and advanced statistical modeling, BKMR, qgcomp, and WQS regression to investigate the interplay of PFAS (PFOA and PFOS), toxic metals (Pb, Cd, and Hg), and essential elements (Se, Mn, and Fe) and their combined impact on CKD.

This study’s socio-demographic, lifestyle, and health characteristics (Table 1 (above)) showed that CDK was prevalent in older individuals (aged 61–80) as compared to younger individuals, people with higher BMI, those with a lower income, and those with comorbidities such as diabetes and hypertension.

The logistic regression analysis (Table 2) showed Cd as a significant risk factor for CKD (OR = 2.16, *p* = 0.023); this aligns with Cd’s well-documented nephrotoxicity and implication in kidney disease. Cd accumulates in the proximal tubules of the kidneys, produces reactive oxygen species (ROS), and induces oxidative stress, mitochondrial dysfunction, and renal tubular atrophy, which are key characteristics of CKD progression [9]. Although Pb was not statistically significant from the logistic regression, its odds ratio showed a positive association (OR = 1.06, *p* = 0.408) with CKD, with its high BKMR PIP of 0.662, as shown in Table 3, confirming its importance. Pb has been associated with decreased kidney function and incidence of CKD due to nephrotoxicity [36].

Hg presented an inverse association with CKD in the logistic regression (OR = 0.92, *p* = 0.408), qgcomp (Figure 7), and WQS (Figure 8); however, it exhibited a non-linear relationship with the BKMR’s univariate–exposure response plot and the highest PIP (0.908), which is a metric that signifies a greater contribution to the outcome variable (CKD). This observation may be attributed to the presence of Se, which has been established to play two key roles in protection against the toxicity of Hg: binding more to Hg through its strong reactive selenol group and serving as an antioxidant to help eliminate the ROS species induced in vivo by Hg [37]. Additionally, the inverse association may reflect dietary co-exposures, as Hg is commonly found in fish that are also rich in omega-3 fatty acids, which have been shown to exert anti-inflammatory and renal-protective effects, potentially confounding or modifying the observed relationship [38].

PFOA was found to be positively associated with CKD (OR = 1.37, *p* = 0.039), as shown in Table 2; this finding is consistent with its documented persistence in renal tissue and potential to disrupt lipid metabolism and tubular function, resulting in adverse effects on kidney function [4]. On the other hand, PFOS was found to have a protective association (OR = 0.94, *p* = 0.013), perhaps because it possesses a more intense serum protein-binding affinity, which may limit it bioaccumulation in the kidneys [39,40].

Se and Mn showed a negative correlation in the correlation matrix heatmap (Figure 1), supporting their function as antioxidants by acting as essential cofactors for glutathione peroxidase (GPx), an enzyme that plays a key role in neutralizing ROS generated by heavy metals such as Cd and Pb as well as antagonizing their effect. This process helps reduce the oxidative stress associated with exposure to these metals. In essence, Se supports the body in countering the detrimental effects of heavy metals by allowing GPx to effectively eliminate harmful free radicals produced during metal-induced oxidative stress [41,42]. However, their lack of statistical significance in logistic regression analysis (Se: OR = 0.998, *p* = 0.791; Mn: OR = 1.03, *p* = 0.512) suggests non-linear or threshold-dependent effects, as depicted in BKMR’s univariate plots (Figure 2). This observation highlights the need for advanced and novel statistical models such as the BKMR, which has the potential to reveal non-linear, non-additive, and overall effects of pollutant mixtures.

The BKMR analysis plot (Figure 2) shows a U-shaped relationship between Hg and CKD, suggesting a non-linear relationship indicating that both high and low levels of Hg may be associated with a risk of CKD. However, higher levels may exhibit an increased risk of the disease than lower levels of Hg. The bivariate exposure–response plot (Figure 3) highlights the effect of the simultaneous interaction between two of the pollutants or mixtures on CKD. Hg showed a consistent pattern (threshold effect) when interacting with all the other exposures. This may imply that no observable effect occurs until a certain level of Hg exposure is reached, but over this level, the risk of CKD increases. This finding is consistent with that of Sabath and Robles-Osorio, who reported that high levels of Cd and Hg or Pb may synergistically impair renal function through a common pathway such as NF-κB activation and ROS production, resulting in oxidative stress [43].

The qgcomp weights (Figure 7) and WQS results (Figure 8) identified Hg and Cd as dominant contributors to CKD risk, which underscores their significant cumulative role as exposures in CKD outcome. This aligns with a study that observed significant association between Pb and Cd with CKD [44]. Another study which is consistent with our findings is that of Choi et al., which found exposure to Cd and Hg is associated with renal tubular damage and other renal damage indicators [45]. The findings for Fe vary across analytical methods, showing no individual association in logistic regression (OR = 1.00, *p* = 0.934), a decreasing effect in the univariate (Figure 2) and bivariate analysis (Figure 3), but contributory effects in BKMR (PIP = 0.67), as shown in Table 3, qgcomp (Figure 7), and WQS (Figure 8), reflecting its dual biological roles. First, it plays a critical role in red blood cell (RBC) production as a significant component of hemoglobin to cater for erythropoietin insufficiency due to kidney damage, signifying its importance. Secondly, excess Fe deposition in renal tubules induces lipid peroxidation through the production of hydroxyl radicals and oxidative stress via the Fenton reaction [46].

Our study’s overall exposure effect shows all exposures, from the 25th to the 75th quantile, with the 50th quantile (median value) serving as the point of comparison (Figure 7). The plot shows an overall decreasing joint effect of the combined PFAS, toxic metals, and essential elements on CKD from the 25th to the 75th quantile compared to when all the pollutants were at their median (50th quantile). This effect is supported by the 95% credible interval, which does not include zero, indicating a high probability of a negative association within this range. This observation might be due to the antagonistic interaction of the essential elements, especially Se and Mn, with the toxic metals, resulting in a decrease in the overall effect of the mixture on CKD. This finding is consistent with the studies conducted by Chen et al. and Wu et al., who found an antagonistic association between Se and Mn with renal function and that their inclusion in the mixture model resulted in a decrease in the overall effect on renal function and CKD [47,48]. Again, our previous study’s BKMR overall effect, which did not include essential elements, exhibited an increasing effect of PFAS and toxic metals on CKD [19].

The limitations of single-pollutant models and linear and logistic regression highlight the need for advanced statistical models in studying environmental mixtures or pollutants and their impact on human health, capturing the non-linear, non-additive, cumulative, and intricate biological interactions.

### 4.2. Limitations

This study relied on cross-sectional data from the NHANES; though nationally representative, it restricts our ability to infer causality. Longitudinal cohort studies tracking the level of pollutants and the decline in renal damage markers, such as eGFR, urinary N-acetyl-β-D-glucosaminidase (NAG), and β2-microglobulin (β2-MG), are needed to establish causality. Again, blood metal levels may not fully capture chronic exposure and could be combined with toe/fingernails or hair sample analysis, which could improve the results and establish temporality and accuracy in the findings.

## 5. Conclusions

CKD remains a significant global health challenge, with environmental pollutants playing a significant role in the disease. This study highlights the complex interplay of PFAS, toxic metals, and essential elements in the disease, while traditional studies have focused mainly on toxic exposures. Cd emerged as a major risk factor, which is consistent with its well-established nephrotoxic activity through oxidative stress and proximal tubular damage, while Hg has a complex, non-linear U-shaped effect relationship. The BKMR model revealed intricate interactions, as indicated in the bivariate plots between Hg and PFOA CKD that traditional linear methods may not capture. Cd, Hg, and PFOA have been established to be persistent in renal tissue and have the potential to disrupt lipid metabolism and induce ROS, oxidative stress, and DNA damage, resulting in adverse effects on kidney function. The findings also reveal the combined effects of toxic metals on CKD, which depicts the real-world scenario where humans are not exposed to a single pollutant but a complex mixture. Essential elements like Se and Mn helped counteract the effect of toxic metals, reducing the overall risk. These findings highlight the need for policies that limit toxic exposures and promote essential nutrient intake in appropriate proportions to protect kidney health, particularly in vulnerable populations. Finally, the use of advanced statistical models like BKMR, qgcomp, and WQS are essential in elucidating these complexities to help in providing targeted public health interventions and strategies.

## Figures and Tables

**Table 1.** (above) Demographic, lifestyle, and health characteristics of participants in this study. (below) Environmental exposure characteristics of participants in this study.

Variable	Total	CKD	Non-CKD	*p*-Value
**Total**	5800	1071(18.47)	4729(81.53)	<0.0001
**Gender**				
Male	2814(48.52)	508(47.43)	2306(48.76)	0.4515
Female	2986(51.48)	563(52.57)	2423(51.24)	
**Age (yrs)**				
0–20	1037(17.88)	93(8.68)	944(19.96)	<0.0001
21–40	1462(25.21)	105(9.80)	1357(28.70)	
41–60	1601(27.60)	248(23.16)	1353(28.61)	
61–80	1700(29.31)	625(58.36)	1075(22.73)	
**BMI**				
<18.5 (Underweight)	199(3.43)	33(3.08)	166(3.51)	<0.0001
18.5–24.9 (Normal)	1616(27.86)	242(22.60)	1374(29.05)	
25–29.9 (Overweight)	1717(29.60)	293(27.36)	1424(30.11)	
≥30 (Obese)	2202(37.97)	480(44.82)	1722(36.41)	
Blank	66(1.14)	23(2.15)	43(0.91)	
**Ethnicity**				
Mexican American	858(14.79)	132(12.32)	726(15.35)	<0.0001
Other Hispanic	535(9.22)	87(8.12)	448(9.47)	
Non-Hispanic White	1988(34.28)	445(41.55)	1543(32.63)	
Non-Hispanic Black	1283(22.12)	231(21.57)	1052(22.25)	
Non-Hispanic Asian	803(13.84)	111(10.36)	692(14.63)	
Other Race—Including Multiracial	333(5.74)	65(6.07)	268(5.67)	
**Annual income**				
<USD 25,000	1534(26.45)	337(31.47)	1197(25.31)	<0.0001
USD 25,000–74,999	2189(37.74)	425(39.68)	1764(37.30)	
USD 75,000–99,999	542(9.34)	79(7.38)	463(9.79)	
≥UDS100,000	1022(17.62)	141(13.17)	881(18.63)	
Missing	513(8.84)	89(8.31)	424(8.97)	
**Alcohol**				
Yes	4244(73.17)	820(76.56)	3424(72.40)	<0.0001
No	509(8.78)	109(10.18)	400(8.46)	
Blank	1047(18.05)	142(13.26)	905(19.14)	
**Smoking**				
Yes	2046(35.28)	465(43.42)	1581(33.43)	<0.0001
No	3017(52.02)	531(49.58)	2486(52.57)	
Missing	737(12.71)	75(7.00)	662(14.00)	
**Hypertension (Taking Medication)**				
Yes	1675(28.88)	604(56.40)	1071(22.65)	<0.0001
No	171(2.95)	22(2.05)	149(3.15)	
Refused	1(0.02)	0(0.00)	1(0.02)	
Did not know	2(0.03)	1(0.09)	1(0.02)	
Missing	3951(68.12)	444(41.46)	3507(74.16)	
**Diabetes**				
Yes	777(13.40)	350(32.68)	427(9.03)	<0.0001
No	4856(83.72)	689(64.33)	4167(88.12)	
Borderline	164(2.83)	32(2.99)	132(2.79)	
Did not know	3(0.05)	0.00)	3(0.06)	
**Kidney Failing**				
Yes	187(3.22)	133(12.42)	54(1.14)	<0.0001
No	4632(79.86)	841(78.52)	3791(80.16)	
Did not know	8(0.14)	6(0.56)	2(0.04)	
Missing	973(16.78)	91(8.50)	882(18.65)	
**eGFR stage**				
Stage 1	3206(55.28)	339(31.65)	2867(60.63)	<0.0001
Stage 2	2117(36.50)	255(23.81)	1862(39.37)	
Stage 3	435(7.50)	435(40.62)	0(0.00)	
Stage 4	33(0.57)	33(3.08)	0(0.00)	
Stage 5	9(0.16)	9(0.84)	0(0.00)	
**ACR (mg/g)**	5.23(8.28, 15.88)	48.05(17.72, 123.30)	6.63(4.57, 10.56)	<0.0001
**SCr (mg/dL)**	0.82(0.68, 0.98)	0.98(0.75, 1.25)	0.80(0.67, 0.93)	<0.0001
Variable	Total	CKD	Non-CKD	p-Value
**PFOA (ng/mL)**	1.37(0.87, 2.07)	1.47(0.87, 2.27)	1.37(0.87, 1.97)	0.072
**PFOS (ng/mL)**	4.20(2.40, 7.80)	5.20(2.70, 9.60)	4.00(2.30, 7.40)	0.0004
**Pb (μg/dL)**	0.76(0.46, 1.30)	1.07(0.66, 1.66)	0.77(0.46, 1.30)	<0.0001
**Cd (μg/L)**	0.22(0.12, 0.42)	0.35(0.20, 0.60)	0.25(0.15, 0.47)	<0.0001
**Hg (μg/L)**	0.51(0.20, 1.12)	0.65(0.34, 1.33)	0.62(0.31, 1.34)	0.1579
**Mn (μg/L)**	9.71(7.75, 12.13)	9.24(7.28, 11.45)	9.57(7.68, 12.03)	<0.0001
**Se (μg/L)**	184.30(169.50, 200.90)	187.90(171.40, 205.80)	188.30(174.3, 204.00)	0.5270
**Fe (μg/dL)**	82.00(61.00, 106.00)	78.00(59.00, 99.00)	83.00(62.00, 108.00)	<0.0001

ACR, albumin creatinine ratio; BMI, body mass index; eGFR, estimated glomerular filtration rate; SCr, serum creatinine; basic characteristics of the study population (n = 5800). CKD, chronic kidney disease; PFOA, perfluorooctanoic acid; PFOS, perfluorooctanesulfonic acid; Cd, cadmium; Pb, lead; Hg, mercury; Se, selenium; Mn, manganese; Fe, iron; HbA1c, glycated hemoglobin; data presented as n (%) or median (25th, 75th percentiles). *p*-values were derived using Mann–Whitney U test for the continuous variables and chi-square test for the category variables. *p*-value is significant at <0.05.

**Table 2.** Logistic regression analysis of exposure variables with CKD.

Exposures	Odds Ratio	95% CI	*p*-Value
**PFOA**	1.370	1.016–1.848	0.039 *
**PFOS**	0.940	0.894–0.987	0.013 *
**Lead**	1.065	0.918–1.235	0.408
**Cadmium**	2.161	1.111–4.203	0.023 *
**Mercury**	0.923	0.765–1.115	0.408
**Selenium**	0.998	0.984–1.012	0.791
**Manganese**	1.032	0.940–1.133	0.512
**Iron**	1.000	0.989–1.010	0.934

*p*-value is significant at * *p* < 0.05; PFOA: perfluorooctanoic acid, PFOS: perfluorooctanesulfonic acid.

**Table 3.** PIPs for the influence of PFAS (PFOA and PFOS), heavy metals (Pb, Cd, and Hg), and essential elements (Se, Mn, and Fe) on CKD.

Exposures	PIP
PFOA	0.701
PFOS	0.612
Lead	0.662
Cadmium	0.668
Mercury	0.908
Selenium	0.616
Manganese	0.754
Iron	0.668

Note: PIPs—posterior inclusion probabilities; PFOA—perfluorooctanoic acid; PFOS—perfluorooctanesulfonic acid.

**Table 4.** Hierarchical BKMR results for posterior inclusion probabilities for the influence of PFAS, toxic metals, and essential elements on CKD.

Exposures	Group	groupPIP	condPIP
PFOA	1	0.251	0.578
PFOS	1	0.251	0.422
Lead	2	0.684	0.029
Cadmium	2	0.684	0.116
Mercury	2	0.684	0.855
Selenium	3	0.702	0.025
Manganese	3	0.702	0.140
Iron	3	0.702	0.835

PFOA: perfluorooctanoic acid, PFOS: perfluorooctanesulfonic acid.

## Data Availability

The NHANES dataset is publicly available online, accessible at https://wwwn.cdc.gov/nchs/nhanes/continuousnhanes/overview.aspx?BeginYear=2017 (accessed on 1 February 2025).
